# Loss of AMPK potentiates inflammation by activating the infammasome in a preclinical mouse model of TBI

**DOI:** 10.1515/nipt-2024-0019

**Published:** 2026-03-02

**Authors:** Mohammad Ejaz Ahmed, Hamid Suhail, Mohammad Nematullah, Benoit Viollet, Shailendra Giri, Abdullah Shafique Ahmad

**Affiliations:** Department of Neurology, Henry FordHealth, 2799 WGrand Blvd,Detroit,MI, 48202, USA; Department of Neurology, Henry Ford Health, Detroit, MI, USA; Department of Neurology, Henry Ford Health, Detroit, MI, USA; Universite Paris Cite, CNRS, Inserm, Institute Cochin, Paris, France; Department of Neurology, Henry Ford Health, Detroit, MI, USA; Department of Neurology, Henry Ford Health, Detroit, MI, USA

**Keywords:** NLRP3 inflammasome, lipopolysaccharides, adenosine monophosphate-activated protein kinase (AMPK), inflammation, functional impairment

## Abstract

**Objectives:**

Traumatic brain injury (TBI) is a major cause of mortality and long-term neurological disabilities. Adenosine monophosphate-activated protein kinase (AMPK), a key cellular energy sensor, plays a critical role in maintaining energy homeostasis. Loss of AMPK phosphorylation following TBI impairs the restoration of cellular energy homeostasis and promotes inflammation. In this study, we investigated whether post-TBI loss of AMPK worsens functional impairments, amplifies inflammation, and exacerbates tissue damage in a mouse model of TBI.

**Methods:**

Adult male C57BL/6 wild-type (WT) and (AMPKα1-KO) mice were subjected to TBI or sham surgery. Behavioral assessments were performed at 24 h post-TBI, followed by mice were anesthetized, and their brains were rapidly collected for histological and biochemical analyses. To further support our findings, mixed glial cells isolated from WT and AMPKα1-KO pups were treated with lipopolysaccharides and interferon-gamma (LI) (0.1 μg/ml LPS and 20 ng/ml IFNg) for 6 h to induce an inflammatory response.

**Results:**

Our results show that TBI reduces AMPK phosphorylation in WT mice and that AMPK loss correlates with worsened behavioral deficits, enhanced NLRP3 inflammasome activation, and elevated levels of pro-inflammatory mediators, including IL-1β. Similarly, AMPKα1-KO glial cells exhibited greater activation of NLRP3 inflammasome and higher expression of pro-inflammatory markers, such as IL-1β, IL-6, TNF-α, iNOS, and Cox 2, compared with WT cells.

**Conclusions:**

Collectively, our results demonstrate that AMPKα1 is a critical endogenous regulator of glial-driven neuroinflammation and secondary tissue damage following TBI. Restoring AMPKα1 activity after TBI may therefore represent a promising therapeutic strategy to attenuate neuroinflammation and limit TBI-associated neurological damage.

## Introduction

Traumatic brain injury (TBI) remains a major contributor to mortality and long-term disability in the United States and worldwide. It results from external mechanical forces that damage brain tissue, triggering a cascade of secondary events, including inflammation and neuronal death. TBI imposes significant emotional and financial burdens on patient families, often requiring long-term care [1, 2]. Despite advances in clinical care, accurately diagnosing TBI severity and implementing effective treatments remain challenging due to the diverse nature of the injury and the complexity of the secondary process. These challenges underscore the urgent need for improved diagnostic tools and therapeutic strategies to enhance outcomes for TBI patients.

Both preclinical and clinical studies have demonstrated that TBI leads to a long-lasting reduction in glucose metabolism, which adversely affects brain function [3-5]. Recent advances in metabolic analysis indicate that pathways regulating energy metabolism play a critical role in controlling neuroinflammation after TBI. AMPK is a serine/threonine kinase that acts as a central cellular energy sensor, and is rapidly activated in response to metabolic stress, hypoxia, excitotoxicity, and altered AMP/ATP or ADP/ATP ratios [6-8] and protect cells against energy depletion by inhibiting anabolic pathways and promoting ATP-generation and increase AMP/ATP ratio [9, 10]. Following TBI, loss of AMPKα1 activity contributes to impaired bioenergetic homeostasis, which in turn amplifies inflammatory response and neuronal vulnerability [11]. Thus, AMPKα1 represents a key molecular link between metabolic dysfunction and neuroinflammation. Following TBI, AMPKα1 activation typically acts to restore cellular energy balance by promoting ATP-generating pathways and regulating energy consumption [12-14].

Recently, AMPK and the NLRP3 inflammasome have attracted significant attention from both preclinical and clinical researchers. The involvement of AMPK in the progression and pathogenesis of various diseases has led to the development of new therapeutic strategies. The inflammasome is a multiprotein complex composed of three key subunits: the sensor molecule NLRP3, the adaptor protein, ASC, and an effector protein, caspase −1. The complex plays a vital role in the innate immune defense by activating inflammatory responses through maturation and release of pro-inflammatory cytokines, such as interleukin-1β (IL-1β), and interleukin-18 (IL-18) [15-18]. Many studies have demonstrated that TBI triggers a complex immune response characterized by the activation of the NLRP3 inflammasome [19, 20]. Previous studies have shown significant increases in the NLRP3, caspase-1, and IL-1β expression at 12 h and 72 h in post-TBI mice [21, 22]. Among the inflammasomes expressed in the brain, NLRP3 promotes the cleavage and activation of caspase-1 and IL-1β, thereby amplifying the inflammatory response following TBI [23-25]. In an animal model of TBI, elevated levels of NLRP3 and the inflammasome complex in the ipsilateral cortex are correlated with long-term functional outcomes [26]. The present study reveals an important role of AMPK/NLRP3 inflammasome-mediated neuroinflammation in TBI. To our knowledge, no previous studies have directly examined the impact of AMPK on NLRP3 inflammasome activation in the controlled cortical impact (CCI) induced mouse model of TBI.

## Materials and methods

### Animals and experimental groups

Adult male C57BL/6 wild-type (WT) and AMPKα1 global knockout (B6/129 background) were used as previously described [27, 28]. These mice were provided by Benoit Viollet (INSERM, Paris) and were generated as reported earlier [29]. The mice were housed and maintained in the animal facility at Henry Ford Health (HFH). Animals were randomly divided into four groups: (i) WT sham (n=10), (ii) WT+TBI (n=10), (iii) AMPKα1-KO sham (n=10), and (iv) AMPKα1-KO+TBI (n=10). All experiments were conducted in accordance with protocol approved by the Henry Ford Health (HFH) Institutional Animal Care and Use Committee (IACUC). Mice were maintained at room temperature (21±1 °C) and 50±5 % humidity under a 12 h dark/light cycle, with *ad libitum* access to food and water.

## Induction of traumatic brain injury

WT and AMPKα1-KO mice underwent either sham or TBI surgery using the controlled cortical impact (CCI) model as previously described [30]. Briefly, mice were anesthetized with 3 % isoflurane for induction, secured in a stereotaxic frame, and maintained under 1.5–2 % isoflurane throughout the procedure using a SomnoSuite low-flow anesthesia system (Kent Scientific Corporation). A craniotomy was performed in the right parietal bone midway between bregma and lambda, 1 mm lateral to the midline, using a hand-held twist drill while leaving the dura intact. The mice were impacted at a velocity of 3 m/s with an 85 ms dwell time and 1.0 mm depth using a 3 mm diameter impactor tip (Pin Point PCI3000 Precision Cortical Impactor, Hatters Instruments). Sham-operated mice underwent the same surgical procedure, without cortical impact. Body temperature was maintained at 37.0±0.5 °C throughout the procedure using a small-animal temperature control system (Kopf Instrument). After surgery, mice were returned to a clean home cage placed on an automatic warming pad (Kent Scientific Corporation).

## Behavioral analysis

### Open field test

The open-field test was used to evaluate locomotor and exploratory activities in rodents [31, 32]. Mice were assessed for the open field test at 24 h post-TBI. Each mouse was placed in the center of the open field apparatus and allowed to explore freely for a 5-min observation period. The time spent in the center zone was recorded and analyzed using the automated ANY-maze video tracking software. The apparatus was cleaned with 70 % ethanol between each test.

### Elevated plus maze test

The elevated plus maze (EPM) test is one of the most widely used methods for assessing anxiety-like behavior in rodents [33]. The EPM test was performed at 24 h post-TBI in mice. Individual mice were placed in the center of the maze and allowed to explore for 5 min. Mouse activity was recorded and analyzed using automated ANY-maze video tracking software. Anxiety-like behavior was evaluated by measuring the time spent in the open arm and the number of open arm entries; mice exhibiting higher anxiety spent less time in the open arm. The EPM apparatus was cleaned with 70 % ethanol between each test.

### Rotarod test

Motor coordination and balance in post-TBI mice were evaluated using a rotarod apparatus, which consists of rotating rods with a rough, solid surface (30 mm in diameter), elevated 30 cm above the floor and divided into four separate compartments, allowing simultaneous testing of up to four mice. Mice were placed on a rotating rod, which initially rotated at 5 revolutions per minute (rpm) and gradually accelerated at a fixed interval to a maximum speed of 20 rpm [34, 35]. Each trial lasted for a maximum duration of 5 min. Each mouse completed three trials, with approximately 30 min of rest between each trial. The average of latency to fall from the three trials was used as the performance score for each mouse.

### Hanging wire test

Grip strength was assessed at 24 h post-TBI in mice as previously described [32]. A wire was stretched between two poles 100 cm apart and 100 cm above the floor. Mice were gently held by the tail and allowed to grasp the wire with their forepaws; the support was then withdrawn, allowing the mouse hang freely from the wire. Each trial was scored on a 5-point scale: 0, fall off; 1, hung by both forepaws; 2, hung by both forepaws with attempts to climb; 3, hung by both forepaws and one or both hind limbs; 4, hung by forepaws with the tail wrapped around the wire; and 5, escape by reaching one of the poles. To prevent injury, a thick, soft mat was placed under the hanging area during testing.

## Western blot analysis

Western blot analysis was performed as previously described [36]. Protein lysates were prepared from the ipsilateral cortical tissue adjacent to the lesion site. This peri-lesion area was selected because it is functionally and metabolically active and undergoes pronounced neuroinflammatory, metabolic, and neuronal changes following TBI. Briefly, 35 μg protein was equally loaded into each well and separated on 4–20 % SDS–PAGE gels. Protein bands were then transferred onto nitrocellulose membranes (Thermo Scientific) and blocked with 5 % nonfat milk for 1 h at room temperature. Membranes were incubated overnight at 4 °C with the appropriate primary antibodies against AMPKα (BS 10344R, Bioss, Woburn, MA), pAMPKα (Thr183/Thr172; 2535S, Cell Signaling), and the NLRP3 (15101, Cell Signaling), IL-1 β (83186S, Cell Signaling), caspase-1(89332T, Cell Signaling). After three washes with TBST, membranes were incubated with the appropriate HRP-conjugated secondary antibody for 2 h at room temperature. The membranes were subsequently washed, and the protein bands were visualized with an enhanced chemiluminescence (ECL) solution (Clarity Western ECL Substrate, 1705062 Bio-Rad Labs, Des Plaines, IL). Quantitative analysis of the bands was performed using ImageJ software (NIH).

## Cresyl violet staining and quantification of lesion size

Coronal brain sections (25 μm thick) were collected using a cryostat at 24 h after TBI. Sections were stained with 0.1 % (*w*/*v*) Cresyl Violet for 5 min, followed by dehydration through graded ethanol concentrations and cleaning in xylene. Lesion volume in each stained section was quantified using ImageJ software (NIH). The mean lesion volume was calculated by subtracting the area of the ipsilateral hemisphere from that of the contralateral hemisphere [37, 38].

## Immunofluorescence staining and quantification

Brains were harvested at 24 h post – TBI and processed for immunofluorescence staining as previously described [36, 39]. Briefly, 25 μm thick brain sections were washed, permeabilized with 0.4 % PBST, and blocked with 0.1 % BSA for 1 h at room temperature. Sections were then incubated overnight at 4 °C with primary antibodies against NeuN (PA5-78499, Invitrogen), MAP2 (MAB3418, Millipore), GFAP (PA5-16291, Thermo Fisher), and Iba1 (016-20001, Wako). The following day, sections were washed 3 times with 0.1 % PBST and incubated for 1 h at room temperature with Alexa Fluor 568/488-conjugated goat anti-mouse/rabbit secondary antibody (1:500; Thermo Scientific Rockford, IL). After washing, sections were mounted on slides with Vectashield mounting medium with 4′,6-diamidino-2-phenyl indole (DAPI) (H-1200; Vector Laboratories, Inc., CA, USA) and covered with a coverslip. Confocal images were acquired using Zeiss AxioTvert 200/Axiovert 200 M microscopes. For quantification, five randomly selected cortical fields per animal were analyzed in four nonadjacent sections spaced approximately 100 μm apart using ImageJ software (NIH).

## Primary brain glial cell culture

The brains of 2- to 3-day-old WT and AMPKα1-KO mouse pups were used to prepare mixed glial cell cultures. We followed the previously published protocol to prepare primary mixed glial cell cultures [40-43]. Briefly, the brains were removed, and the cerebral cortices were isolated and cleared of blood vessels and meninges. Cerebral tissues were mechanically dissociated into a single cell suspension using a Pasteur pipette and resuspended in complete medium (DMEM 4.5 g/L glucose) supplemented with 10 % FBS and 10 mg/mL antibiotics. Cells were seeded into poly-d-lysine-coated 75 cm^2^ flasks and maintained at 37 °C in a humidified 5 % CO_2_ incubator. After 24 h, the media were replaced with fresh complete DMEM and subsequently changed twice per week. Culture was maintained for 12–14 days until they reached full confluence and were ready for dissociation.

## Brain glial cell stimulation

Glial cells isolated from WT and AMPKα1-KO mice were seeded at a density of 50 × 10^4^ cells per well in 24-well plates containing DMEM supplemented with 10 % fetal bovine serum (FBS). Cells were incubated overnight at 37 °C in a humidified atmosphere with 5 % CO_2_. The following day, the culture medium was replaced with serum-free DMEM, and the cells were serum-starved for 2 h. After serum starvation, cells were stimulated with LI (0.1 μg/ml LPS and 20 ng/ml IFNγ) to induce an inflammatory response. Culture supernatants were collected for cytokine ELISA, and cell lysates were processed for qPCR and immunoblot analyses.

## RNA extraction, cDNA synthesis, and quantitative PCR

After stimulation with LI, WT, and AMPKα1-KO glia cells were harvested at 6 h, and total RNA was extracted using Triazole followed by purification with a RNeasy Kit (Qiagen) according to the manufacturer’s instructions. cDNA was synthesized from 1 μg of total RNA using an iScript reverse transcription cDNA synthesis kit (Bio-Rad). Primer sets were selected from a publicly available database and purchased from Integrated DNA Technology (IDT, Coralville, IA, USA). Quantitative real-time PCR was performed using iTaq Universal SYBR Green Supermix (Bio-Rad). Thermal cycling amplification conditions were as follows: polymerase activation at 95 °C for 3 min, followed by 40 cycles of amplification at 95 °C for 30 s and 60 °C for 30 s. Expression of the ribosomal protein L27 housekeeping gene was used for normalization, and relative gene expression was calculated using the (^^Ct) method with CFX Maestro Software.

## Cytokine analysis by enzyme-linked immunosorbent assay (ELISA)

To analyze cytokine levels, isolated glial cells were stimulated with LI (0.1 μg/ml LPS and 20 ng/ml IFNg). After 6 h of stimulation, the culture plates were washed three times with warm serum-free media, and the supernatant was collected to measure inflammatory cytokine production by ELISA.

## Statistical analysis

Data are represented as mean±SEM. Statistical analyses were performed using GraphPad Prism 9 with either oneway analyses of variance (ANOVA) or Student’s *t*-test, as appropriate. For multiple group comparison, the Tukey post-hoc test was applied. A 95 % confidence level (p<0.05) was considered statistically significant.

## Results

### TBI causes loss of AMPKα1 phosphorylation in the mouse brain

**Figure 1:**
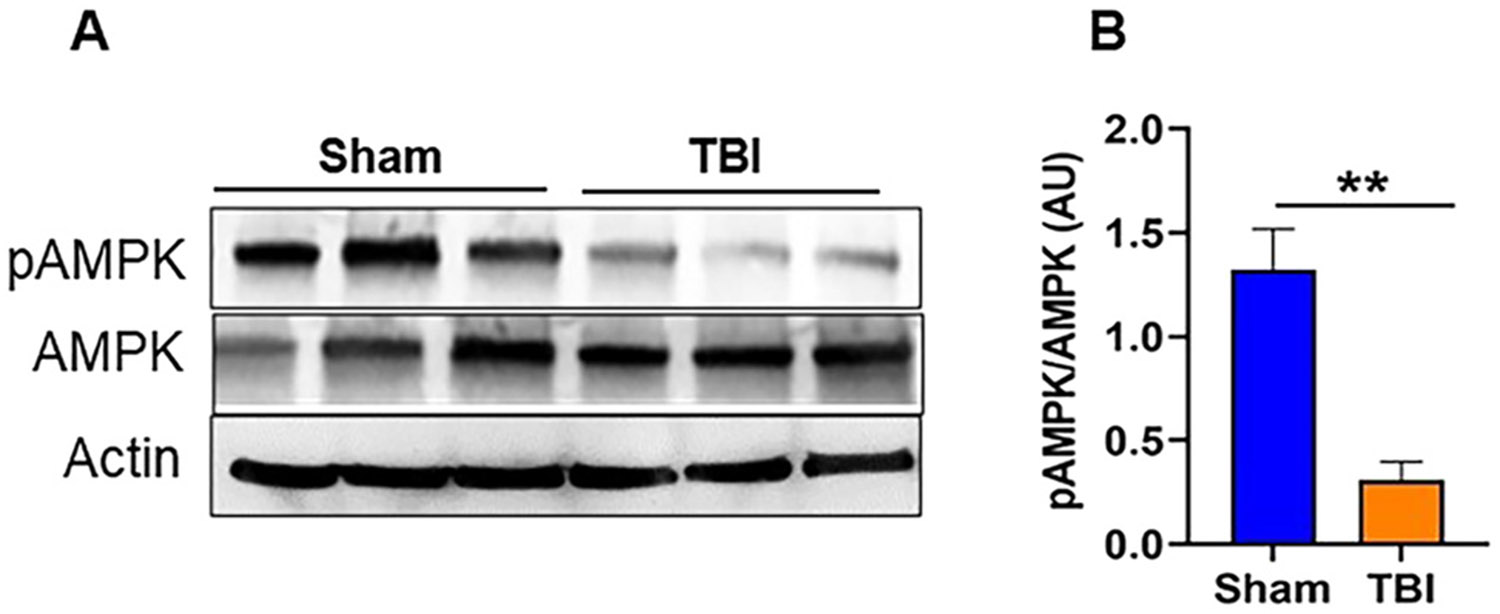
Loss of AMPKα1 phosphorylation following TBI in mice. (A) Representative western blot images showing a loss of AMPKα1 phosphorylation at 24 h post-TBI in WT mice. (B) Quantitative analysis of AMPKα1 levels in the sham and TBI mice. Data are expressed as the mean±SEM (n=3/group); **p<0.01.

WT mouse brains were harvested 24 h after TBI, and Western blot analyses were performed to assess AMPKα1 phosphorylation at Thr172. Representative immunoblots and corresponding quantification (Figure 1A and B) show a significant reduction in phospho-AMPKα1 levels in post-TBI mice compared with sham-operated controls. These results suggest that TBI leads to a decrease in AMPKα1 activity, which may contribute to an overall suppression of AMPKα1 signaling in the injured brain. This initial observation of loss of AMPKα1 phosphorylation in WT mice provides the rationale for further examination of whether loss of AMPKα1 directly contributes to post-TBI neuropathology. To address this question, we extended our studies in AMPK-α1-KO mice and glial cells to investigate the functional and inflammatory consequences of AMPK-α1 loss following TBI in mice.

### AMPKα1 deficiency worsened functional deficits in mice following TBI

**Figure 2:**
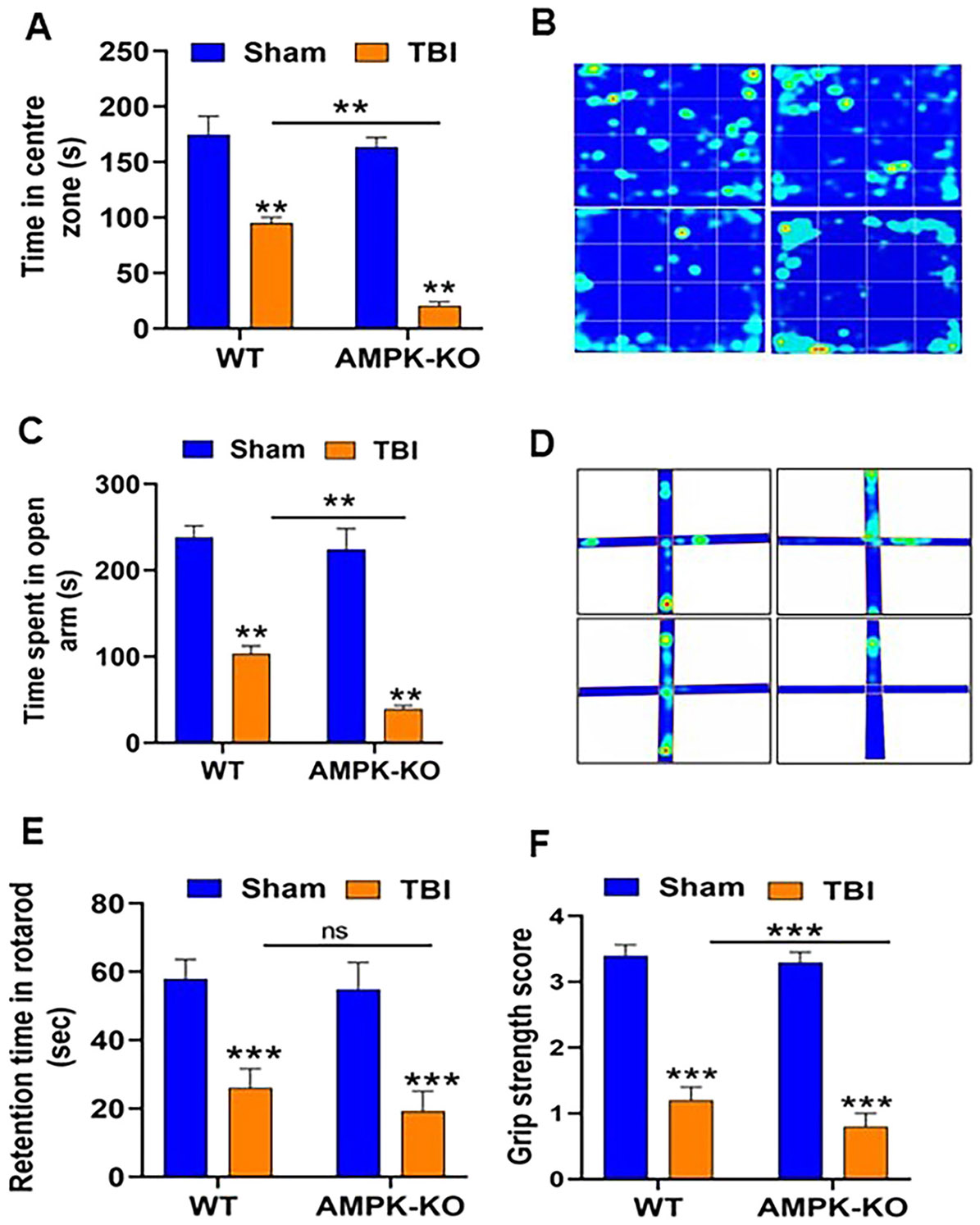
AMPKα1 deficiency worsened functional impairments in TBI mice. The functional impairments were assessed at 24 h post-TBI. (A) Open field test showed that AMPKα1-KO TBI mice spent significantly less time in the central zone compared with WT mice, indicating increased anxiety-like behavior. (C) The elevated plus maze test further confirmed anxiety-like behavior with AMPKα1-KO TBI mice spending significantly less time in the open arm than WT TBI mice. (E) The rotarod test demonstrated reduced motor coordination and balance in AMPKα1-KO TBI mice, as they spent less time on the rotating rod compared to WT TBI mice. (F) Grip strength (hanging wire) test revealed impaired dexterity in AMPKα1-KO TBI mice relative to WT TBI mice. (B, D) Representative heat maps from the open field and elevated plus maze tests show distinct activity patterns between WT and AMPKα1-KO TBI mice. Data are represented as means±SEM (n=10/group); **p<0.01, ***p<0.001.

To investigate the impact of AMPKα1 loss on functional outcomes after TBI, WT and AMPKα1-KO mice were assessed for behavioral impairments at 24 h post-injury. Spontaneous locomotor activity was evaluated using the open-field test by measuring the time spent in the center of the field. AMPKα1-KO TBI mice spent significantly less time in the central zone than WT TBI mice (Figure 2A and B), indicating increased anxiety-like behavior. Anxiety-like behavior was further assessed using the elevated plus maze at 24 h post-TBI. AMPKα1-KO mice spent less time in the open arm compared with WT mice, confirming that AMPKα1 deficiency exacerbates anxiety-like behavior after TBI mice (Figure 2C and D). Motor coordination and grip strength were evaluated using the rotarod and hanging wire tests, respectively (Figure 2E and F). AMPKα1-KO TBI mice exhibited significantly more severe motor deficits compared with WT TBI mice. Collectively, these findings demonstrate that loss of AMPKα1 worsens functional impairment following TBI.

### Loss of AMPKα1 increases lesion size in TBI mice

**Figure 3:**
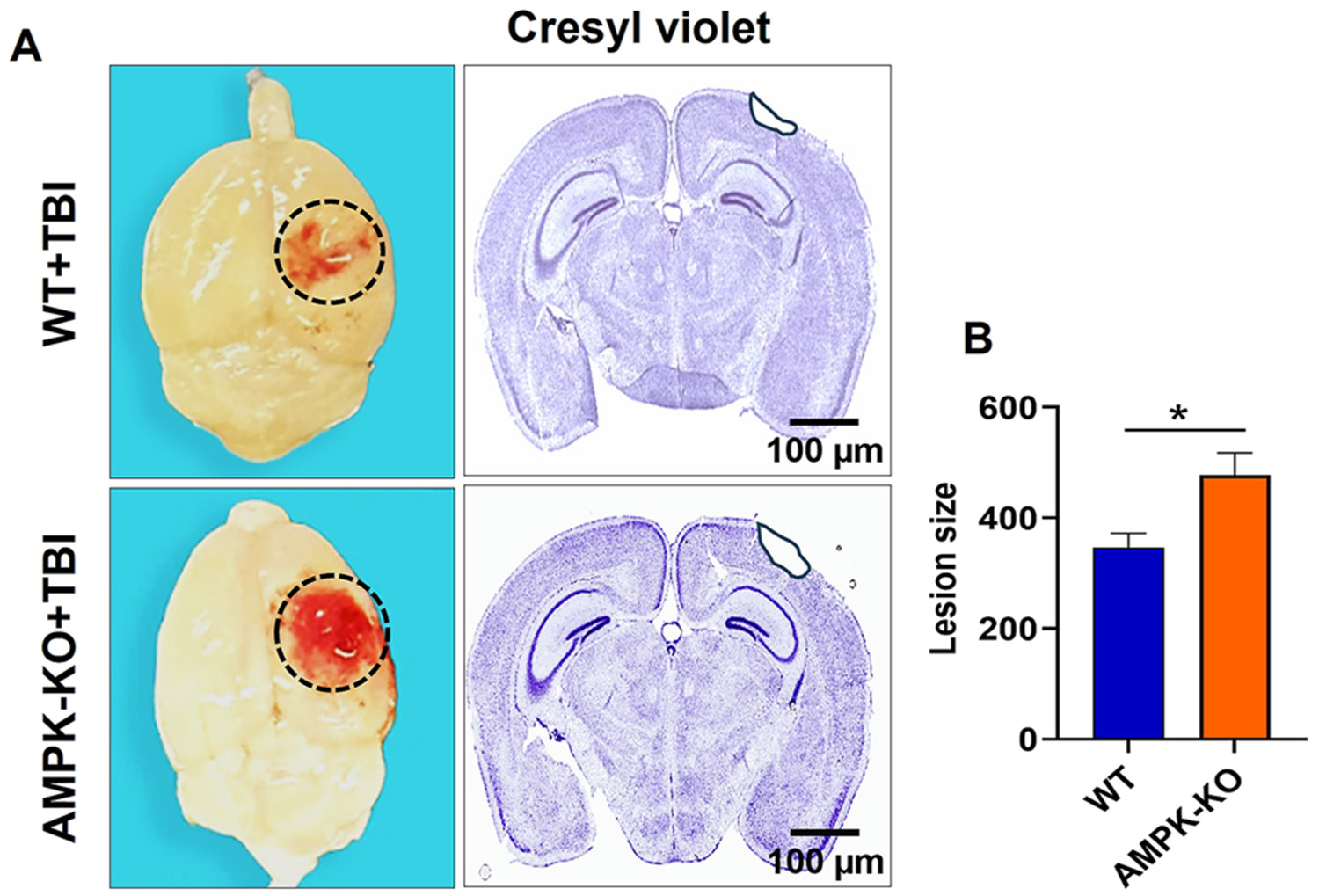
Effect of AMPKα1 loss on histological and anatomical changes in the brain after TBI. (A) Blood clots and tissue damage at the impact site were assessed in WT and AMPKα1-KO mice at 24 h post-TBI. Representative cresyl violet-stained brain sections show the lesion site highlighted in black. (B) Quantitative analysis of lesion size reveals significantly larger lesions in AMPKα1-KO mice compared with WT mice. Data are expressed as mean±SEM (n=5 mice/group); *p<0.05. Scale bar=100 μm.

The impact of AMPK loss on intracranial hemorrhage and lesion size was evaluated at 24 h post-TBI in WT and AMPKα1-KO mice. Representative whole-brain images revealed a marked increase in intracranial hemorrhage in AMPKα1-KO mice compared with WT mice (Figure 3A). Quantitative analysis confirmed that AMPKα1-KO mice exhibited significantly greater intracranial hemorrhage than WT TBI mice. In addition, lesion size in the ipsilateral hemisphere was significantly larger in AMPKα1-KO mice than in WT mice (Figure 3B). These findings indicate that the loss of AMPK exacerbates brain injury after TBI, resulting in increased bleeding and more extensive tissue damage.

### Loss of AMPKα exacerbates TBI-induced neuronal damage in the mouse brain

**Figure 4:**
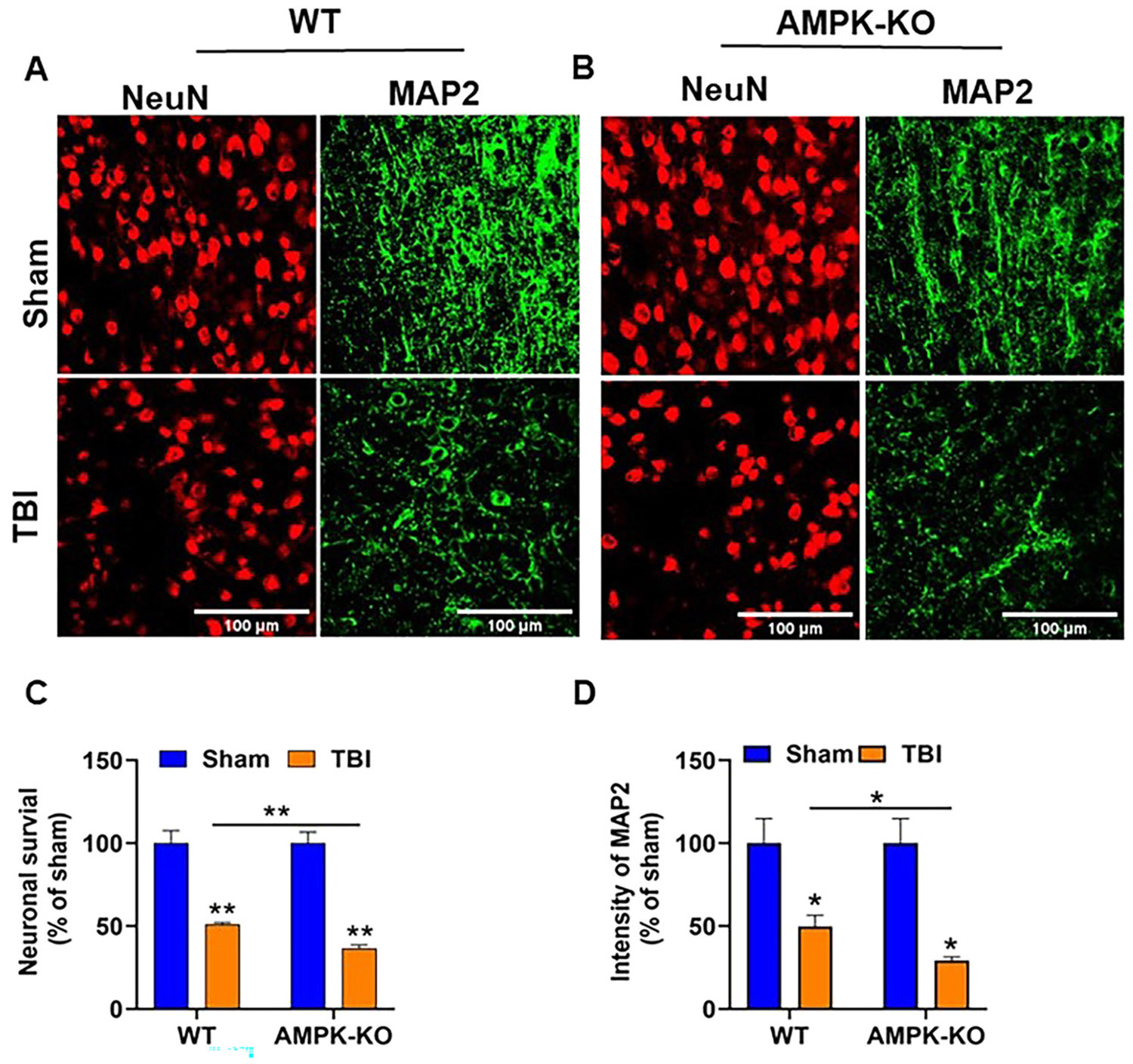
The absence of AMPKα1 enhances neuronal injury after TBI. (A, B) Representative immunofluorescence images of MAP2 (green) and NeuN (red) in the ipsilateral cortex of WT and AMPKα1-KO mice at 24 h post-TBI. (C, D) Quantitative analysis shows that both WT and AMPKα1-KO sham groups display strong MAP2 intensity and NeuN expression, indicating preserved neuronal integrity. In contrast, AMPKα1-KO TBI mice exhibit greater neuronal loss. Data are expressed as the mean±SEM (n=5 mice/group); *p<0.05, **p<0.01, Scale bar=100 μm.

We next examined the impact of AMPKα1 loss on TBI-induced neuronal loss using immunofluorescence staining. Brain sections from WT and AMPKα1-KO mice were stained with the neuron-specific markers microtubule-associated protein 2 (MAP2, green) and (NeuN, red). MAP2 primarily labels the neuronal cytoskeleton, including dendrites, whereas NeuN marks neuronal nuclei and perinuclear cytoplasm (Figure 4A and B). Compared with WT TBI mice, AMPKα1-KO TBI mice showed significantly reduced MAP2 immunoreactivity and NeuN staining (Figure 4C and D), indicating exacerbated neuronal loss at 24 h post-TBI.

### AMPKα1 deficiency exacerbates NLRP3 inflammasome-mediated neuroinflammation in TBI mouse brains

**Figure 5:**
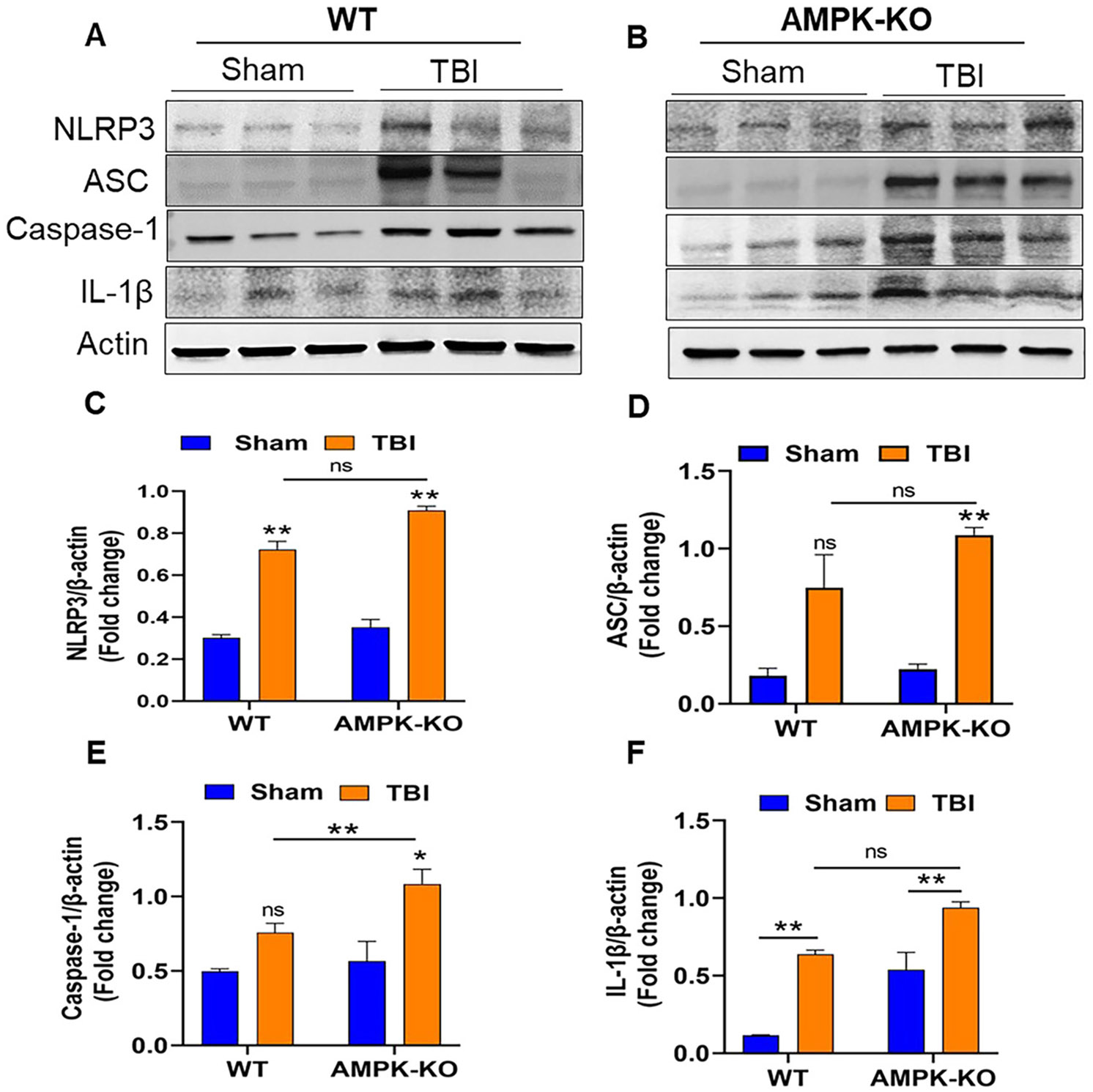
AMPKα1 deficiency potentiates inflammasome activation in the brain following TBI. (A, B) Western blot images showing expression of inflammasome markers NLRP3, ASC, caspase-1, and IL-1β, (C–F) quantitative analysis of the blots demonstrates that AMPKα1-KO TBI mice exhibited increased expression of inflammasome components compared with WT TBI mice. Band intensities were normalized by actin. Data are presented as the means±SEM (n=3/group); *p<0.05, **p<0.01, ns=not significant.

Inflammasomes play a crucial role in regulating the inflammatory response following TBI. Representative western blot images from AMPKα1-KO TBI mice exhibited significantly higher expression of NLRP3, ASC, caspase-1, and IL-1β, compared with WT TBI mice (Figure 5A and B). Quantitative analysis confirmed elevated levels of these inflammasome markers in AMPKα1-KO mice relative to WT TBI mice (Figure 5C-F). Notably, during TBI, loss of AMPKα1 was associated with an increase in NLRP3 inflammasome activation, suggesting a potential inverse regulatory relationship between AMPKα1 activity and inflammasome signaling.

**Figure 6:**
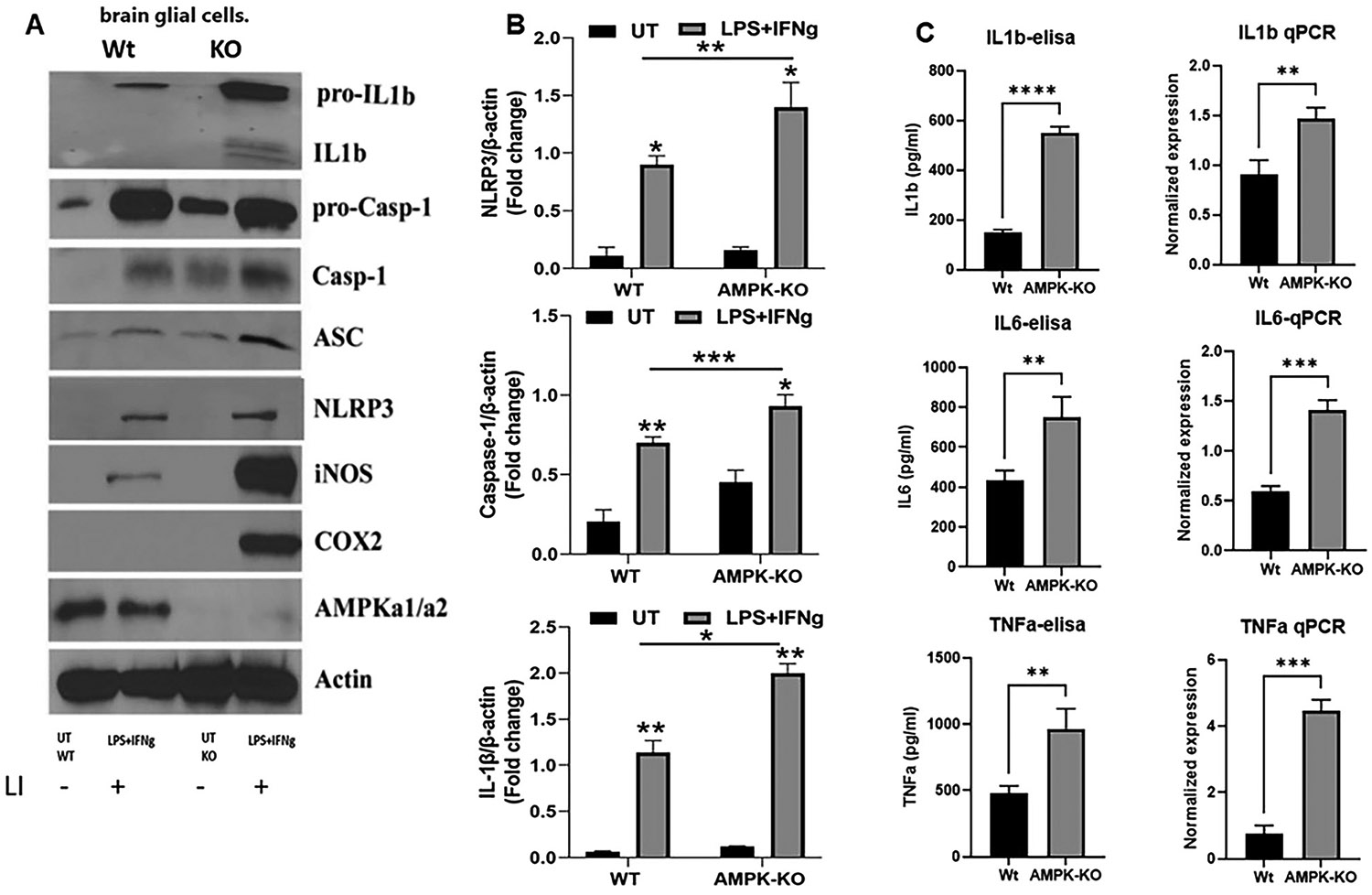
Loss of AMPKα1 in brain glial cells promotes hyperinflammation and inflammasome activation. WT and AMPKα1-KO mice glial cells were treated with LPS/IFNg (0.1 μg/20 ng/ml) to induce inflammation. (A) Cell lysates were analyzed by immunoblotting for various inflammatory mediators and inflammasome proteins using specific antibodies as described in the methods section. Band intensities of key inflammasome proteins were quantified (B). (C) The cell supernatant was used for the ELISA detection of IL1b, IL6, and TNFα, and the cells were processed for mRNA detection by qPCR (n=4). The blots are representative of two experiments with similar observations. The data are represented as the means±SEM (n=3/groups); **p<0.01, ***p<0.001.

To further explore this relationship, glial cells isolated from WT and AMPKα1-KO pups were stimulated with inflammatory agents LI (0.1 μg/ml LPS and 20 ng/ml IFNγ) to assess inflammation and inflammasome activation. Western blot, qPCR, and ELISA were used to measure these markers. AMPKα1 deficiency led to increased levels of inflammatory mediators, including iNOS and Cox2, accompanied by enhanced inflammasome activity, as indicated by elevated NLRP3, ASC, cleaved caspase-1, and IL-1β levels compared to WT glial cells (Figure 6A). Quantitative analysis of key inflammasome components NLRP3, caspase-1 and 1L-1β revealed significantly higher band intensity in AMPKα1-KO cells than in WT cells (Figure 6B). Similarly, inflammatory cytokines, including IL-1β, IL-6, and TNFα, were significantly elevated in AMPK-KO glial cells under inflammatory conditions (Figure 6C). These results indicate that AMPKα1 functions as a negative regulator of inflammation and inflammasome activation.

### AMPKα1 deficiency increases TBI-induced gliosis in the mouse brain

**Figure 7:**
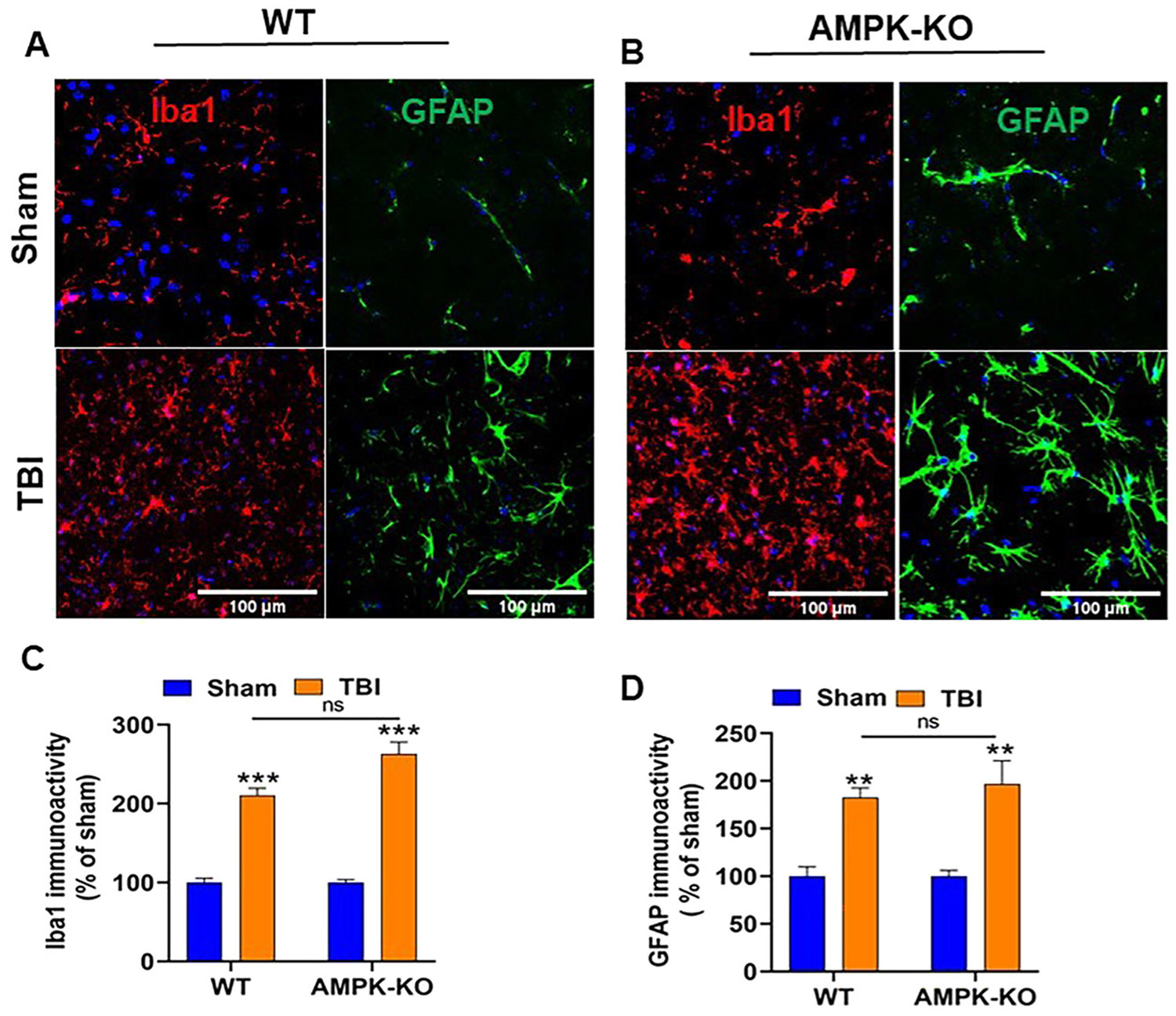
Effect of AMPKα1 deficiency on reactive astrogliosis following TBI. The expression of astrocytes and microglia in WT and AMPKα1-KO mouse brains was assessed using immunofluorescence staining. (A, B) Representative confocal images show increased GFAP (green, astrocyte markers) and Iba1 (red, microglial markers) expression in AMPK-KO mice compared with WT mice at 24 h post-TBI. Scale bar=100 μm. (C, D) Quantitative analysis of GFAP and Iba1 immunoreactivity in the brains of WT and AMPK-KO mice. Data are represented as the mean ±SEM from 4–5 sections per brain (n=6 mice/group); **p<0.01, ***p<0.001, ns=not significant, scale bar=100 μm.

Immunofluorescence staining was performed to assess microglial and astrocyte activation in the ipsilateral cortex at 24 h post-TBI. Results showed increased immunoreactivity of GFAP (green), an astrocyte marker, and Iba1 (red), a microglial marker, in AMPKα1-KO mice compared with WT mice (Figure 7A and B). Quantitative analysis indicated elevated GFAP and Iba1 expression in AMPKα1-KO TBI mice; The difference between WT and AMPKα1-K OTBI mice was not statistically significant (Figure 7C and D).

## Discussion

Our study provides novel insight into the role of AMPK in the context of TBI and associated inflammation. We observed a significant reduction in AMPKα1 activity, as evidenced by decreased phosphorylation at Thr172 following TBI in WT mice. This reduction in AMPKα1 phosphorylation was associated with worsened functional outcomes and increased neuroinflammation characterized by the upregulation of NLRP3 inflammasome and pro-inflammatory cytokines. Furthermore, we demonstrated that reduced AMPK levels exacerbate post-TBI inflammation, which is accompanied by aggravated functional deficits, increased brain tissue damage, and enhanced neuronal loss. Collectively, these findings reveal a previously unexplored role of AMPK in modulating inflammation and brain damage following TBI.

TBI induces distinct metabolic responses in the brain, characterized by an acute rise in metabolic activity and increased glucose utilization that lasts for up to 30 min post-injury [4]. However, this initial hypermetabolic phase is followed by a prolonged period of hypometabolism, which can be detected as early as 6 h post-injury and persists for days to weeks thereafter [3, 4, 44]. In our study, we initially examined the phosphorylation status of AMPK in the ipsilateral cortex 24 h post-TBI in WT TBI mice. Our results revealed that TBI significantly reduced AMPKα1 level in WT mice, as evidenced by decreased phosphorylation at Thr172. Based on this initial observation in WT animals, we next employed AMPKα1-KO mice to further confirm the role of AMPK in TBI pathology. Consistent with our hypothesis, AMPKα1 deficiency markedly exacerbated brain injury, as evidenced by reduced MAP2 and NeuN expression in the ipsilateral cortex adjacent to the lesion site. Loss of MAP2 staining indicates dendritic destabilization and cytoskeleton disruption, whereas a decrease in NeuN staining suggests increased neuronal loss or impaired neuronal activity in AMPKα1-KO mice compared with WT TBI mice. The reduction in MAP2 and NeuN expression was primarily observed in the ipsilateral region. Future studies examining these markers in additional brain regions, including contralateral and distal regions, will provide a more comprehensive understanding of neuronal survival and dendritic stability following brain injury. These finding confirming a critical role of AMPKα1 in modulating the pathological processes associated with TBI.

Previous studies have shown that activation of the NLRP3 inflammasome promotes inflammation and exacerbates brain damage in a mouse model of brain injury [45-47]. Conversely, accumulating evidence indicates that AMPKα1 plays a key role in suppressing inflammasome expression, thereby attenuating brain damage [48-50]. Together, these findings suggest that AMPKα1-mediated modulation of inflammation may represent a key mechanism for mitigating TBI-induced neuroinflammation and resulting neurological damage. Consistent with this concept, we found that loss of AMPKα1 led to the increased activation of key inflammatory components, including NLRP3, ASC, caspase-1, and IL-1β, at 24 h post-injury. Collectively, these results underscore the therapeutic potential of targeting AMPK to reduce post-TBI inflammation and associated neurological damage.

The brain’s innate immune system plays a key role in the response to TBI. Resident microglia and astrocytes become activated upon sensing danger signals and initiate an inflammatory cascade. Proteins associated with the activation of these cells are commonly used as markers in TBI [51, 52]. Our results demonstrated that loss of AMPKα1 following TBI led to increased expression of GFAP and Iba1 in AMPKα1-KO TBI mice compared to WT TBI mice. These findings highlight an important role of AMPK in regulating gliosis and inflammation after TBI.

## Conclusions

Our findings support the hypothesis that loss of AMPKα1 following TBI is associated with worsened behavioral outcomes and enhanced inflammasome activation. These results suggest that activation of AMPKα1 may represent a promising therapeutic strategy to reduce neuroinflammation and improve functional recovery after injury. Accordingly, future studies investigating selective AMPK activators may provide a valuable approach for mitigating inflammation and promoting recovery following TBI.

## Limitations of this study

This study primarily focused on assessing the inflammatory role of AMPKα1 in a preclinical mouse model of TBI. We did not explore the potential therapeutic benefits of AMPKα1 activation following injury. Moreover, our analyses were limited to male mice at 24 h post-injury, examining AMPKα1 reduction and subsequent inflammasome activation. Further studies are needed to exmaine the inflammatory role of AMPKα1 in female mice at the same time point. It is also essential to examine the long-term effect of TBI on AMPKα1 signaling and brain hypometabolism to better understand the translational relevance and progression of injury-related changes. Additionally targeting AMPK activation in glial or neuronal cells using specific AMPK activators may help clarify the mechanism underlying AMPKα1 loss and NLRP3 Inflammasome activation following TBI. These investigations may provide valuable insight for developing targeted therapies aimed at modulating AMPK activity to improve outcomes following TBI.

## References

[1] Barlow KM, Esser MJ, Veidt M, Boyd R. Melatonin as a treatment after traumatic brain injury: a systematic review and meta-analysis of the pre-clinical and clinical literature. J Neurotrauma 2019;36:523–37. 29901413 10.1089/neu.2018.5752

[2] Maas AIR, Menon DK, Adelson PD, Andelic N, Bell MJ, Belli A, Traumatic brain injury: integrated approaches to improve prevention, clinical care, and research. Lancet Neurol 2017;16:987–1048. 29122524 10.1016/S1474-4422(17)30371-X

[3] Hovda DA, Lee S, Smith M, Von Stuck S, Bergsneider M, Kelly D, The neurochemical and metabolic cascade following brain injury: moving from animal models to man. J Neurotrauma 1995;12:903–6. 8594218 10.1089/neu.1995.12.903

[4] Yoshino A, Hovda DA, Kawamata T, Katayama Y, Becker DP. Dynamic changes in local cerebral glucose utilization following cerebral conclusion in rats: evidence of a hyper- and subsequent hypometabolic state. Brain Res 1991;561:106–19. 1797338 10.1016/0006-8993(91)90755-k

[5] Vespa P, Bergsneider M, Hattori N, Wu HM, Huang SC, Martin NA, Metabolic crisis without brain ischemia is common after traumatic brain injury: a combined microdialysis and positron emission tomography study. J Cerebr Blood Flow Metabol 2005;25:763–74. 10.1038/sj.jcbfm.9600073 PMC4347944 15716852

[6] Gusarova GA, Trejo HE, Dada LA, Briva A, Welch LC, Hamanaka RB, Hypoxia leads to Na, K-ATPase downregulation via Ca(2+) release-activated Ca(2+) channels and AMPK activation. Mol Cell Biol 2011;31:3546–56. 21730292 10.1128/MCB.05114-11 PMC3165547

[7] Mungai PT, Waypa GB, Jairaman A, Prakriya M, Dokic D, Ball MK, Hypoxia triggers AMPK activation through reactive oxygen species-mediated activation of calcium release-activated calcium channels. Mol Cell Biol 2011;31:3531–45. 21670147 10.1128/MCB.05124-11 PMC3165558

[8] Concannon CG, Tuffy LP, Weisová P, Bonner HP, Dávila D, Bonner C, AMP kinase-mediated activation of the BH3-only protein Bim couples energy depletion to stress-induced apoptosis. J Cell Biol 2010;189:83–94. 20351066 10.1083/jcb.200909166 PMC2854380

[9] Karagounis LG, Hawley JA. The 5′ adenosine monophosphate-activated protein kinase: regulating the ebb and flow of cellular energetics. Int J Biochem Cell Biol 2009;41:2360–3. 19628050 10.1016/j.biocel.2009.07.004

[10] Peixoto CA, Oliveira WH, Araujo S, Nunes AKS. AMPK activation: role in the signaling pathways of neuroinflammation and neurodegeneration. Exp Neurol 2017;298:31–41. 28844606 10.1016/j.expneurol.2017.08.013

[11] Hill JL, Kobori N, Zhao J, Rozas NS, Hylin MJ, Moore AN, Traumatic brain injury decreases AMP-activated protein kinase activity and pharmacological enhancement of its activity improves cognitive outcome. J Neurochem 2016;139:106–19. 27379837 10.1111/jnc.13726 PMC5037010

[12] Hardie DG, Ross FA, Hawley SA. AMPK: a nutrient and energy sensor that maintains energy homeostasis. Nat Rev Mol Cell Biol 2012;13:251–62. 22436748 10.1038/nrm3311 PMC5726489

[13] Hurley RL, Anderson KA, Franzone JM, Kemp BE, Means AR, Witters LA. The Ca2+/calmodulin-dependent protein kinase kinases are AMP-activated protein kinase kinases. J Biol Chem 2005;280:29060–6. 15980064 10.1074/jbc.M503824200

[14] Weisova P, Concannon CG, Devocelle M, Prehn JH, Ward MW. Regulation of glucose transporter 3 surface expression by the AMP-activated protein kinase mediates tolerance to glutamate excitation in neurons. J Neurosci 2009;29:2997–3008. 19261894 10.1523/JNEUROSCI.0354-09.2009 PMC6666202

[15] O’Neill LA, Hardie DG. Metabolism of inflammation limited by AMPK and pseudo-starvation. Nature 2013;493:346–55. 23325217 10.1038/nature11862

[16] Steinberg GR, Schertzer JD. AMPK promotes macrophage fatty acid oxidative metabolism to mitigate inflammation: implications for diabetes and cardiovascular disease. Immunol Cell Biol 2014;92:340–5. 24638063 10.1038/icb.2014.11

[17] Baroja-Mazo A, Martín-Sánchez F, Gomez AI, Martínez CM, Amores-Iniesta J, Compan V, The NLRP3 inflammasome is released as a particulate danger signal that amplifies the inflammatory response. Nat Immunol 2014;15:738–48. 24952504 10.1038/ni.2919

[18] Schroder K, Zhou R, Tschopp J. The NLRP3 inflammasome: a sensor for metabolic danger? Science 2010;327:296–300. 20075245 10.1126/science.1184003

[19] Hang CH, Chen G, Shi JX, Zhang X, Li JS. Cortical expression of nuclear factor kappaB after human brain contusion. Brain Res 2006;1109:14–21. 16857176 10.1016/j.brainres.2006.06.045

[20] Martinon F, Burns K, Tschopp J. The inflammasome: a molecular platform triggering activation of inflammatory caspases and processing of proIL-beta. Mol Cell 2002;10:417–26. 12191486 10.1016/s1097-2765(02)00599-3

[21] Ma J, Xiao W, Wang J, Wu J, Ren J, Hou J, Propofol inhibits NLRP3 inflammasome and attenuates blast-induced traumatic brain injury in rats. Inflammation 2016;39:2094–103. 27696022 10.1007/s10753-016-0446-8

[22] Wei X, Hu CC, Zhang YL, Yao SL, Mao WK. Telmisartan reduced cerebral edema by inhibiting NLRP3 inflammasome in mice with cold brain injury. J Huazhong Univ Sci Technol Med Sci 2016;36:576–83. 10.1007/s11596-016-1628-1 27465336

[23] Qian H, Li Q, Shi W. Hyperbaric oxygen alleviates the activation of NLRP-3-inflammasomes in traumatic brain injury. Mol Med Rep 2017;16:3922–8. 29067455 10.3892/mmr.2017.7079 PMC5646971

[24] Wallisch JS, Simon DW, Bayır H, Bell MJ, Kochanek PM, Clark RSB. Cerebrospinal fluid NLRP3 is increased after severe traumatic brain injury in infants and children. Neurocritical Care 2017;27:44–50. 28181102 10.1007/s12028-017-0378-7 PMC5680164

[25] Frugier T, Morganti-Kossmann MC, O’Reilly D, McLean CA. In situ detection of inflammatory mediators in post mortem human brain tissue after traumatic injury. J Neurotrauma 2010;27:497–507. 20030565 10.1089/neu.2009.1120

[26] Adamczak S, Dale G, de Rivero Vaccari JP, Bullock MR, Dietrich WD, Keane RW. Inflammasome proteins in cerebrospinal fluid of brain-injured patients as biomarkers of functional outcome: clinical article. J Neurosurg 2012;117:1119–25. 23061392 10.3171/2012.9.JNS12815 PMC3576729

[27] Nath N, Khan M, Rattan R, Mangalam A, Makkar RS, Meester C, Loss of AMPK exacerbates experimental autoimmune encephalomyelitis disease severity. Biochem Biophys Res Commun 2009;386:16–20. 19486896 10.1016/j.bbrc.2009.05.106 PMC2976596

[28] Mangalam AK, Rattan R, Suhail H, Singh J, Hoda MN, Deshpande M, AMP-activated protein kinase suppresses autoimmune central nervous system disease by regulating M1-type macrophage-Th17 axis. J Immunol 2016;197:747–60. 27354217 10.4049/jimmunol.1501549

[29] Jorgensen SB, Viollet B, Andreelli F, Frøsig C, Birk JB, Schjerling P, Knockout of the alpha2 but not alpha1 5′-AMP-activated protein kinase isoform abolishes 5-aminoimidazole-4-carboxamide-1-beta-4-ribofuranosidebut not contraction-induced glucose uptake in skeletal muscle. J Biol Chem 2004;279:1070–9. 14573616 10.1074/jbc.M306205200

[30] Braun M, Vaibhav K, Saad N, Fatima S, Brann DW, Vender JR, Activation of myeloid TLR4 mediates T lymphocyte polarization after traumatic brain injury. J Immunol 2017;198:3615 – 26. 28341672 10.4049/jimmunol.1601948 PMC5417078

[31] Ahmed ME, Dong Y, Lu Y, Tucker D, Wang R, Zhang Q. Beneficial effects of a CaMKIIalpha inhibitor TatCN21 peptide in global cerebral ischemia. J Mol Neurosci 2017;61:42–51. 27604243 10.1007/s12031-016-0830-8 PMC5219940

[32] Vaibhav K, Braun M, Alverson K, Khodadadi H, Kutiyanawalla A, Ward A, Neutrophil extracellular traps exacerbate neurological deficits after traumatic brain injury. Sci Adv 2020;6:eaax8847. 32523980 10.1126/sciadv.aax8847 PMC7259928

[33] Haller J, Aliczki M, Gyimesine Pelczer K. Classical and novel approaches to the preclinical testing of anxiolytics: a critical evaluation. Neurosci Biobehav Rev 2013;37:2318–30. 22981935 10.1016/j.neubiorev.2012.09.001

[34] Ahmed ME, Selvakumar GP, Kempuraj D, Raikwar SP, Thangavel R, Bazley K, Glia maturation factor (GMF) regulates microglial expression phenotypes and the associated neurological deficits in a mouse model of traumatic brain injury. Mol Neurobiol 2020;57:4438–50. 32737763 10.1007/s12035-020-02040-y

[35] Yang SH, Gustafson J, Gangidine M, Stepien D, Schuster R, Pritts TA, A murine model of mild traumatic brain injury exhibiting cognitive and motor deficits. J Surg Res 2013;184:981–8. 23622728 10.1016/j.jss.2013.03.075 PMC4073786

[36] Selvakumar GP, Ahmed ME, Iyer SS, Thangavel R, Kempuraj D, Raikwar SP, Absence of glia maturation factor protects from axonal injury and motor behavioral impairments after traumatic brain injury. Exp Neurobiol 2020;29:230–48. 32565489 10.5607/en20017 PMC7344375

[37] Vaibhav K, Shrivastava P, Khan A, Javed H, Tabassum R, Ahmed ME, Azadirachta indica mitigates behavioral impairments, oxidative damage, histological alterations and apoptosis in focal cerebral ischemia-reperfusion model of rats. Neurol Sci 2013;34:1321–30. 23187787 10.1007/s10072-012-1238-z

[38] Ahmed ME, Tucker D, Dong Y, Lu Y, Zhao N, Wang R, Methylene blue promotes cortical neurogenesis and ameliorates behavioral deficit after photothrombotic stroke in rats. Neuroscience 2016;336:39–48. 27590267 10.1016/j.neuroscience.2016.08.036 PMC5056839

[39] Zhao N, Zhuo X, Lu Y, Dong Y, Ahmed ME, Tucker D, Intranasal delivery of a caspase-1 inhibitor in the treatment of global cerebral ischemia. Mol Neurobiol 2017;54:4936–52. 27520275 10.1007/s12035-016-0034-9 PMC5305804

[40] Giri S, Khan M, Nath N, Singh I, Singh AK. The role of AMPK in psychosine mediated effects on oligodendrocytes and astrocytes: implication for Krabbe disease. J Neurochem 2008;105:1820–33. 18248608 10.1111/j.1471-4159.2008.05279.x PMC2673995

[41] Giri S, Khan M, Rattan R, Singh I, Singh AK. Krabbe disease: psychosine-mediated activation of phospholipase A2 in oligodendrocyte cell death. J Lipid Res 2006;47:1478–92. 16645197 10.1194/jlr.M600084-JLR200

[42] Giri S, Rattan R, Singh AK, Singh I. The 15-deoxy-delta12,14-prostaglandin J2 inhibits the inflammatory response in primary rat astrocytes via down-regulating multiple steps in phosphatidylinositol 3-kinase-Akt-NF-kappaB-p300 pathway independent of peroxisome proliferator-activated receptor gamma. J Immunol 2004;173:5196 – 208. 15470065 10.4049/jimmunol.173.8.5196

[43] Suhail H, Nematullah M, Rashid F, Sajad M, Fatma M, Singh J, An early glycolysis burst in microglia regulates mitochondrial dysfunction in oligodendrocytes under neuroinflammation. iScience 2023;26:107921. 37841597 10.1016/j.isci.2023.107921 PMC10568429

[44] Giza CC, Hovda DA. The neurometabolic cascade of concussion. J Athl Train 2001;36:228–35. 12937489 PMC155411

[45] Irrera N, Pizzino G, Calò M, Pallio G, Mannino F, Famà F, Lack of the Nlrp3 inflammasome improves mice recovery following traumatic brain injury. Front Pharmacol 2017;8:459. 28769794 10.3389/fphar.2017.00459 PMC5509758

[46] Liu HD, Li W, Chen ZR, Hu YC, Zhang DD, Shen W, Expression of the NLRP3 inflammasome in cerebral cortex after traumatic brain injury in a rat model. Neurochem Res 2013;38:2072–83. 23892989 10.1007/s11064-013-1115-z

[47] Xu X, Yin D, Ren H, Gao W, Li F, Sun D, Selective NLRP3 inflammasome inhibitor reduces neuroinflammation and improves long-term neurological outcomes in a murine model of traumatic brain injury. Neurobiol Dis 2018;117:15–27. 29859317 10.1016/j.nbd.2018.05.016

[48] Yang F, Qin Y, Wang Y, Meng S, Xian H, Che H, Metformin inhibits the NLRP3 inflammasome via AMPK/mTOR-dependent effects in diabetic cardiomyopathy. Int J Biol Sci 2019;15:1010–19. 31182921 10.7150/ijbs.29680 PMC6535781

[49] Liu GJ, Tao T, Wang H, Zhou Y, Gao X, Gao YY, Functions of resolvin D1-ALX/FPR2 receptor interaction in the hemoglobin-induced microglial inflammatory response and neuronal injury. J Neuroinflammation 2020;17:239. 32795323 10.1186/s12974-020-01918-x PMC7429751

[50] Xu W, Li T, Gao L, Zheng J, Yan J, Zhang J, Apelin-13/APJ system attenuates early brain injury via suppression of endoplasmic reticulum stress-associated TXNIP/NLRP3 inflammasome activation and oxidative stress in a AMPK-dependent manner after subarachnoid hemorrhage in rats. J Neuroinflammation 2019;16:247. 31791369 10.1186/s12974-019-1620-3 PMC6889224

[51] Hernandez-Ontiveros DG, Tajiri N, Acosta S, Giunta B, Tan J, Borlongan CV. Microglia activation as a biomarker for traumatic brain injury. Front Neurol 2013;4:30. 23531681 10.3389/fneur.2013.00030 PMC3607801

[52] Diaz-Arrastia R, Wang KK, Papa L, Sorani MD, Yue JK, Puccio AM, Acute biomarkers of traumatic brain injury: relationship between plasma levels of ubiquitin C-terminal hydrolase-L1 and glial fibrillary acidic protein. J Neurotrauma 2014;31:19–25. 23865516 10.1089/neu.2013.3040 PMC3880090

